# Effects of Coffee Consumption on Insulin Resistance and Sensitivity: A Meta-Analysis

**DOI:** 10.3390/nu13113976

**Published:** 2021-11-08

**Authors:** Su-Min Moon, Min-Jin Joo, Young-Seo Lee, Myeong-Gyu Kim

**Affiliations:** 1 College of Pharmacy, Ewha Womans University, Seoul 03760, Korea; your6596@naver.com (S.-M.M.); minjin960812@naver.com (M.-J.J.); sylvia0616@hanmail.net (Y.-S.L.); 2 Graduate School of Pharmaceutical Sciences, Ewha Womans University, Seoul 03760, Korea

**Keywords:** coffee, meta-analysis, insulin resistance, insulin sensitivity

## Abstract

Coffee is widely consumed worldwide and impacts glucose metabolism. After a previous meta-analysis that evaluated the effects of coffee consumption on insulin resistance and sensitivity, additional randomized controlled trials (RCTs) were conducted. This meta-analysis aimed to evaluate the effects of coffee consumption on insulin resistance or sensitivity. We selected RCTs that evaluated the effects of coffee consumption for seven days or more on insulin sensitivity or resistance using surrogate indices (homeostasis model assessment for insulin resistance (HOMA-IR) and Matsuda index). The fixed-effects or random-effects model was used according to heterogeneity. Four studies with 268 participants were analyzed in this meta-analysis. Coffee consumption significantly decreased HOMA-IR compared to control (mean difference (MD) = −0.13; 95% CI = −0.24–−0.03; *p*-value = 0.01). However, the significance was not maintained in the sensitivity analysis (MD = −0.04; 95% CI = −0.18–0.10; *p*-value = 0.55) after excluding data from the healthy, young, normal-weight group. Matsuda index was not significantly different between coffee and control groups (standardized mean difference (SMD) = −0.33; 95% CI = −0.70–0.03; *p*-value = 0.08). In conclusion, long-term coffee consumption has a nonsignificant effect on insulin resistance and sensitivity. More studies evaluating the effects of coffee consumption in the healthy, young, and normal-weight individuals are needed.

## 1. Introduction

Coffee has become one of the most famous drinks today and is increasingly consumed globally. Coffee contains various bioactive ingredients that can positively or negatively affect the human body [1,2]. Coffee consumption is inversely associated with total mortality [3,4,5], endometrial cancer [6], colon cancer [7], hepatic cancer [8], prostate cancer [9], and chronic liver disease [10].

Diabetes mellitus is a rapidly growing global problem with large social, health, and economic consequences [11]. Many cohort and nested case-control studies have been conducted on the association between coffee consumption and type 2 diabetes. A meta-analysis of 30 epidemiologic studies found that drinking a cup of coffee daily reduces the risk of diabetes by 6% (relative risk (RR) = 0.94; 95% confidence interval (CI), 0.93–0.95) [12]. The effect size between caffeinated coffee and decaffeinated coffee consumption did not differ significantly (RR = 0.93; 95% CI = 0.90–0.96 vs. RR = 0.94; 95% CI = 0.90–0.98) [12]. The association between coffee consumption and diabetes has been shown only in epidemiologic studies; randomized clinical trials (RCTs) directly verifying this association are limited. Instead, RCTs have studied the effects of coffee consumption on glucose and insulin levels. In a recent meta-analysis, coffee and decaffeinated coffee consumption did not significantly affect fasting blood glucose concentration (mean difference (MD) = 1.34 mg/dL; 95% CI = −0.52–3.20 mg/dL and MD = 5.28 mg/dL; 95% CI = −5.34–15.91 mg/dL, respectively) [13]. Moreover, the effects of coffee and decaffeinated coffee at 2-h post-75-g glucose load plasma glucose concentration were not significant (MD = −23.99 mg/dL; 95% CI = −63.78–15.81 mg/dL and MD = 12.27 mg/dL; 95% CI = −8.52–33.07 mg/dL) [13]. Coffee significantly altered fasting insulin concentration (MD = 1.1 μIU/mL; 95% CI = 0.17–2.03 μIU/mL) [13]. However, measurements of these concentrations have limitations in evaluating insulin sensitivity and resistance.

The hyperinsulinemic-euglycemic clamp is the gold standard for assessing insulin sensitivity in humans [14]. However, this method is not suitable for use in clinical practice because it is a time-consuming, labor-intensive, and expensive method, and requires skilled examiners. Several surrogate indices (e.g., homeostasis model assessment (HOMA), quantitative insulin sensitivity check index (QUICKI), Matsuda, McAuley, Belfiore, Cederholm, Avignon, and Stumvoll indexes) have been developed as alternative measures of insulin resistance or sensitivity [15,16].

Few studies have assessed the effects of coffee consumption on insulin sensitivity/resistance indices. Until 2017, only two studies were included in meta-analysis and systematic reviews [13,17]. Newer studies have been conducted since then, which makes it essential to evaluate the influence of coffee consumption on insulin resistance or sensitivity through a meta-analysis. This meta-analysis aimed to evaluate the effects of coffee consumption on insulin resistance or sensitivity.

## 2. Materials and Methods

### 2.1. Literature Search

This study followed a pre-planned protocol and adhered to the Preferred Reporting Items for Systematic reviews and Meta-Analyses checklist. A literature search was conducted using PubMed and Embase on 26 July 2021. Search queries were a combination of population, intervention, comparison, and outcome (PICO) terms in Table 1. RCT search filters were used. There was no restriction on publication language. We searched Google Scholar and reference lists of relevant literature (e.g., recent review articles and selected articles in this meta-analysis) for inclusion.

### 2.2. Study Selection

Studies were included if they (1) were RCTs, (2) included participants consuming coffee (caffeinated or decaffeinated) for 7 days or more, and (3) evaluated the effects of coffee on insulin sensitivity or resistance using surrogate indices. Studies on green coffee bean extracts or caffeine capsules were excluded.

First, the title and abstracts of articles were screened for eligibility. Second, full-text articles were reviewed and selected for this meta-analysis. Two researchers independently conducted this process, and any discrepancy was resolved through discussion or arbitration with a third researcher. EndNote X9 (Clarivate Analytics, Philadelphia, PA, USA) was used for managing the articles.

### 2.3. Data Extraction and Quality Assessment

The following data were extracted from the eligible studies: (1) authors and publication year, (2) study country, (3) study design, (4) number, age, weight or BMI, health condition of participants, and (5) insulin sensitivity or resistance measures (baseline, final, or change). HOMA for insulin resistance (HOMA-IR) and Matsuda index were reported in more than one study, and meta-analysis was possible. If the study results were presented only in a graph, an online data extraction tool (https://ij.imjoy.io/, accessed on 27 August 2021) was used to extract data from the graph.

The quality of the studies was evaluated using the Risk of Bias 2 (RoB 2) tool (The Cochrane Collaboration, Copenhagen, Denmark) that included bias arising from the randomization process, bias due to deviations from intended interventions, bias due to missing outcome data, bias in the measurement of the outcome, and bias in the selection of the reported result. For each trial, researchers evaluated each addressed item as follows: low risk, some concerns, or high risk.

### 2.4. Data Synthesis and Statistical Analysis

The meta-analysis was conducted using RevMan version 5 software (The Cochrane Collaboration, Copenhagen, Denmark). Data were entered in the form of MD or standardized mean difference (SMD) with standard error (SE). MD represented the difference in the amount of change before and after treatment between the intervention and control groups. SE was calculated by dividing the standard deviation (SD) by the square root of the sample size. The combined SDs for crossover study and for change before and after treatment were calculated as the square root of [SD_A_^2^ + SD_B_^2^ − (2 × r × SD_A_ × SD_B_)], assuming a correlation coefficient (r) = 0.5. For studies using log-transformed data, after converting to the original data, new mean values and SD values were obtained as follows:

$${\bar{x}}_{i}^{\prime }=\;\exp\left({\bar{z}}_{i}+\frac{s_{z,i}^{2}}{2}\right)$$

$$s_{x,i}^{\prime }=\sqrt{\left(\exp\left(s_{z,i}^{2}\right)-1\right)\exp\left(2{\bar{z}}_{i}+s_{z,i}^{2}\right)}$$

where ${\bar{x}}_{i}$ is mean of raw measurements. ${\bar{z}}_{i}$ and $s_{z}$ are the mean and SD of log-transformed measurements [18].

Heterogeneity between studies was assessed using Higgins’ I^2^ and Cochran’s Q tests. We defined a considerable heterogeneity as I^2^ > 75% or a *p*-value of the Q test < 0.05. The inverse variance method with the fixed-effects or random-effects model was used to calculate a pooled estimate. If there was considerable heterogeneity, the random-effects model was used; and if not, the fixed-effects model was used. The results are displayed as forest plots.

A subgroup analysis according to caffeine content (caffeinated or decaffeinated) and a sensitivity analysis were conducted. Publication bias was not assessed because of the small number of included studies.

## 3. Results

### 3.1. Selected Studies

**Figure 1:**
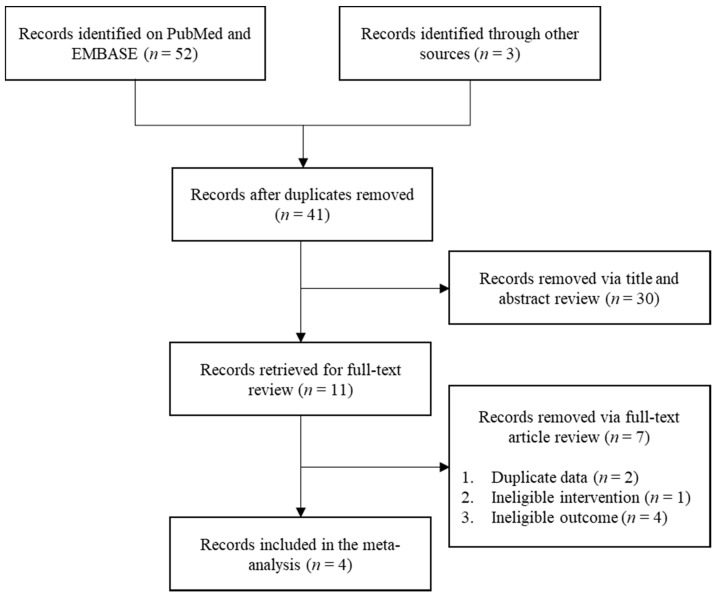
Flowchart of the meta-analysis.

**Figure 2:**
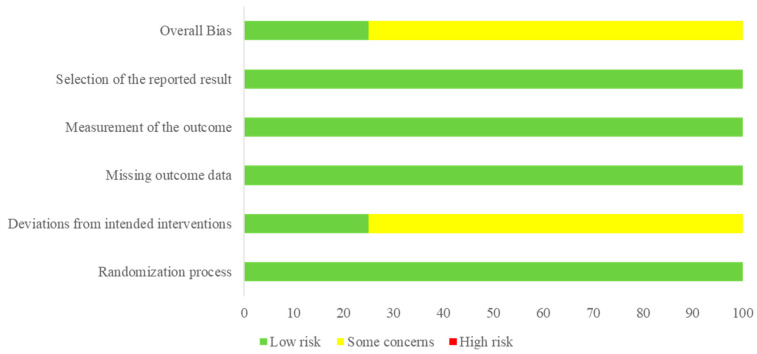
Quality of included studies.

Figure 1 shows the flowchart of this meta-analysis. We identified 41 studies after removing duplicates through a literature search. Through two steps of study selection, four studies were finally included in the meta-analysis [19,20,21,22]. Table 2 summarizes the characteristics of the selected studies. One study by Ohnaka et al. [20] included prediabetic or diabetic adults (fasting plasma glucose 100–140 mg/dL). Other studies included non-diabetic adults. Figure 2 shows the quality of included studies. Three studies exhibited a score of “some concerns” and one study had a score of “low risk”.

### 3.2. HOMA-IR

The four studies with 268 participants reported the effect of coffee consumption on HOMA-IR. One study by Sarriá et al. [21] reported a significant decrease in HOMA-IR after coffee consumption in the normocholesterolemic subgroup but not in the hypercholesterolemic subgroup. Other studies showed no significant difference between the coffee consumption and control groups.

**Figure 3:**
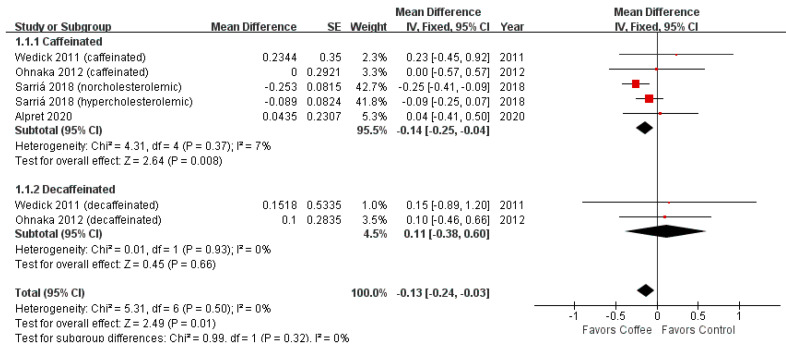
Effects of coffee on HOMA-IR.

Figure 3 shows the forest plot of HOMA-IR. Coffee consumption significantly decreased HOMA-IR compared to the control (MD = −0.13; 95% CI = −0.24–−0.03; *p*-value = 0.01). The significant difference was maintained in the caffeinated coffee subgroup (MD = −0.14; 95% CI = −0.25–−0.04; *p*-value = 0.01).

**Figure 4:**
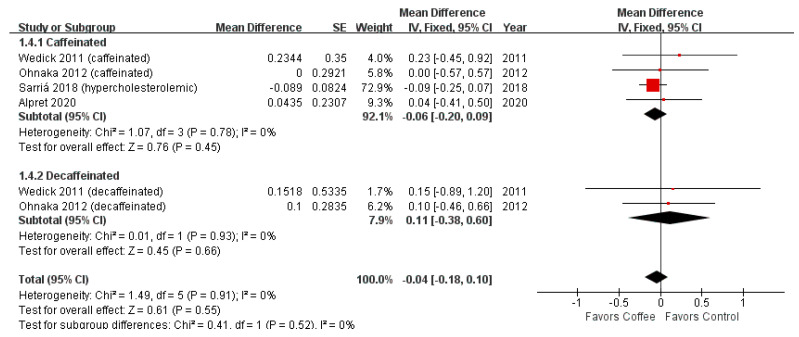
Effects of coffee on HOMA-IR (Sensitivity analysis).

However, sensitivity analysis revealed no significant difference between the coffee consumption and control groups after excluding the results of the normocholesterolemic subgroup in the study by Sarriá et al. [21] (MD = −0.04; 95% CI = −0.18–0.10; *p*-value = 0.55) (Figure 4).

### 3.3. Matsuda Index

The Matsuda index was reported in two studies with 90 participants. These studies assessed both caffeinated and decaffeinated coffee consumption and obtained nonsignificant results.

**Figure 5:**
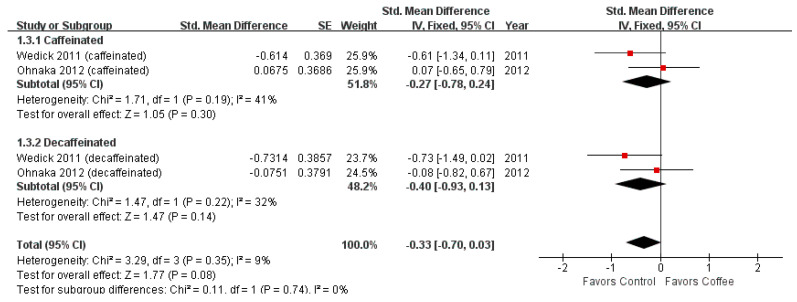
Effects of coffee on Matsuda index.

Figure 5 shows the forest plot of the Matsuda index. The Matsuda index was not significantly different between the coffee consumption and control groups (SMD = −0.33; 95% CI = −0.70–0.03; *p*-value = 0.08). This non-significance remained unchanged in subgroup analysis. The effect size of the caffeinated coffee consumption group was −0.27 (95% CI = −0.78–0.24) and that of the decaffeinated coffee consumption group was −0.40 (95% CI = −0.93–0.13).

## 4. Discussion

This meta-analysis evaluated the effects of coffee consumption on HOMA-IR and Matsuda index by analyzing four RCTs. HOMA-IR is used in many studies as a tool for evaluating insulin resistance and mainly reflects liver insulin resistance [23,24]. This index was more reliable to assess insulin resistance than the fasting glucose/insulin ratio and was an independent predictor of cardiovascular disease [25,26]. Matsuda index is a simple index of whole-body insulin sensitivity including liver and muscle [24,27]. Other surrogate indices, except for HOMA-IR and Matsuda, were rarely used in coffee studies. Although not included in the search terms, insulin resistance indices using C-peptide levels (e.g., clamp-like index (CLIX), C-peptide immunoreactivity insulin resistance (CPR-IR)) exist [28,29]. However, these indices have not been used previously to assess the effects of coffee.

A previous meta-analysis reported no significant effect of coffee consumption on HOMA-IR relative to the control by analyzing two RCTs [13]. Our study, which analyzed two more studies, showed that coffee consumption significantly decreased HOMA-IR. However, the robustness of the result was not warranted. The significance of our result was driven by one significant result from a large-weighted study. The weight of the crossover design is generally larger than that of parallel design in a meta-analysis [30]. The characteristics of the group showing significant results were different from those of other studies. They were young adults (aged 18–45 years), had a normal weight (BMI < 25 kg/m^2^), and did not have any metabolic syndrome including hypercholesterolemia. In such a population, the possibility of coffee consumption lowering insulin resistance cannot be ruled out. However, it is difficult to conclude that coffee consumption reduces insulin resistance from one study. Moreover, coffee consumption did not significantly affect the Matsuda index.

Although not included in this meta-analysis, in one non-RCT, HOMA-IR was 3.93, 4.10, and 4.22 in subgroups that consumed zero, four, and eight cups of coffee daily, respectively [31]. The difference was not significant, and other markers of glucose metabolism also were not significantly different [31]. Some studies reported HOMA for β-cell function (HOMA-B) as an indicator of insulin resistance. There was no significant difference in HOMA-B between the coffee consumption and placebo groups in a study by Alperet et al. [19]. Mansour et al. conducted an RCT that administered two main coffee components, caffeine and chlorogenic acid, to patients with non-alcoholic fatty liver disease and type 2 diabetes [32]. HOMA-IR between chlorogenic acid plus caffeine, chlorogenic acid, caffeine, and placebo did not differ significantly [32].

Previous studies showed that caffeine can lower insulin sensitivity and increase insulin resistance and glucose concentration [33,34,35]. MacKenzie et al. conducted a randomized crossover trial and found that 400 mg of caffeine (equivalent to two cups of coffee) per day decreases insulin sensitivity in young adults [35]. The mechanism of caffeine’s effects on glucose metabolism has not been fully revealed, but several have been suggested. Caffeine inhibits glucose uptake and glycogen synthase activity in the skeletal muscle by competitively blocking adenosine receptors [36]. Other mechanisms include increased levels of epinephrine and free fatty acids that can increase insulin resistance after caffeine intake [35,36]. However, the nonsignificant effects of coffee on insulin resistance and sensitivity in the present meta-analysis might be due to other ingredients in coffee that may negate the effects of caffeine on insulin resistance and sensitivity [37]. Chlorogenic acid reduced glucose concentrations, and its metabolite, quinides, increased insulin sensitivity in rats [38]. Chlorogenic acid may competitively inhibit glucose absorption in the intestine and reduce hepatic glucose output through glucose-6-phosphatase inhibition [38].

This study has several limitations. First, the effects of coffee consumption for more than 24 weeks were not evaluated. This is a limitation of RCTs compared to epidemiologic studies, but a controlled setting (e.g., pre-defined coffee intake, randomly assigned participants) reduces bias and outweighs the disadvantages of an RCT design. Second, the small number of studies limited the evaluation of effect size according to the amount of coffee consumption or caffeine content and the characteristics of the participants through meta-regression. Since coffee is widely consumed and caffeine is addictive, it is difficult to conduct RCTs controlling coffee consumption. Moreover, controlling several confounding variables that affect glucose metabolism (e.g., diet composition and exercise) is difficult. Third, there was heterogeneity in study designs (parallel and crossover). Some researchers recommend combining the results for each research design, which was not done in this meta-analysis due to the small number of included studies.

## 5. Conclusions

Long-term caffeinated or decaffeinated coffee consumption does not negatively affect insulin resistance or sensitivity. There is no need to restrict coffee intake in non-diabetic, prediabetic, and diabetic individuals for fear of insulin resistance. In addition, more studies evaluating the effects of coffee consumption in healthy, young, and normal-weight individuals are needed.

## Figures and Tables

**Table 1:** Population, intervention, comparison, and outcome (PICO) search strategy.

PICO	Keywords
Population	Not defined
Intervention	Coffee
Comparison	Not defined
Outcome	Insulin sensitivity, insulin resistance, homeostasis model assessment (HOMA), quantitative insulin sensitivity check index (QUICKI), Matsuda, McAuley, Belfiore, Cederholm, Avignon, Stumvoll, Gutt

**Table 2:** Characteristics of selected studies.

Author (Year)	Country	Design	Duration	Sample Size	Population	Intervention	Control	Outcome
Alperet (2020) [19]	Singapore	Parallel	24 weeks	126	Non-diabetic, non-smokers, aged 35–69 years, overweight (BMI 22.5–35.4 kg/m^2^), habitual coffee drinkers (≥1 cup/day), not insulin sensitive (HOMA-IR ≥ 1.30), not-having other illnesses that could affect study outcomes	Instant coffee beverage (73.7% of a nondairy creamer) four cups per day. Contained 30 kcal per cup with 0.96 g/100 g of caffeine. Sweeteners (caloric or artificial) or milk was permitted	Coffee-like placebo beverage four cups per day. Contained 30 kcal per cup	HOMA-IR
Sarriá (2018) [21]	Spain	Crossover	8 weeks per period	52	Men and women aged 18–45 years, BMI < 25 kg/m^2^, non-smokers, non-vegetarian, non-pregnant women, not-having vitamins or dietary supplements, not-having taken antibiotics 6 months before, not suffering chronic disorders, apart from hypercholesterolemia	2 g serving of the coffee blend dissolved in 200 mL of hot water, without milk or sugar three times per day. The daily consumption of hydroxycinnamic acids and methylxanthines was 510.6 and 123 mg (121.2 mg was caffeine), respectively	Control drink consisting of water or an isotonic caffeine- and polyphenol-free drink three times per day	HOMA-IR
Ohnaka (2012) [20]	Japan	Parallel	16 weeks	45	Men aged 40–64 years, BMI 25–30 kg/m^2^, fasting plasma glucose 100–140 mg/dL	One cup/glass of coffee using one spoonful (1.2–1.3 g) of instant coffee five times per day (caffeinated or decaffeinated). With mineral water one 500 mL bottle. Either hot or ice coffee was permitted, but coffee was drunk without sugar, milk, or any other additives	Two 500-mL bottles per day	HOMA-IR, Matsuda index
Wedick (2011) [22]	United States	Parallel	8 weeks	45	Non-diabetic, regular coffee consumers (≥2 cups/day), non-smokers, aged ≥ 18 years, overweight (BMI 25–35 kg/m^2^), but otherwise healthy	2 g portions of instant coffee with 6 ounces of boiling water five times per day (caffeinated (345 mg caffeine per day) or decaffeinated). A non-caloric sweetener or a non-dairy creamer was permitted	6 ounce glass of water five times per day	HOMA-IR, Matsuda index

BMI, body mass index; HOMA-IR, homeostasis model assessment for insulin resistance.

## Data Availability

The data presented in this study are available on request from the corresponding author.
